# Searching for the Secret of Stickiness: How Biofilms Adhere to Surfaces

**DOI:** 10.3389/fmicb.2021.686793

**Published:** 2021-07-08

**Authors:** Zhaowei Jiang, Thomas Nero, Sampriti Mukherjee, Rich Olson, Jing Yan

**Affiliations:** ^1^Department of Molecular, Cellular and Developmental Biology, Yale University, New Haven, CT, United States; ^2^Department of Molecular Genetics and Cell Biology, University of Chicago, Chicago, IL, United States; ^3^Department of Molecular Biology and Biochemistry, Molecular Biophysics Program, Wesleyan University, Middletown, CT, United States; ^4^Quantitative Biology Institute, Yale University, New Haven, CT, United States

**Keywords:** adhesion, bacteria, biofilm, extracellular matrix, biomechanics, infection, pathogenesis

## Abstract

Bacterial biofilms are communities of cells enclosed in an extracellular polymeric matrix in which cells adhere to each other and to foreign surfaces. The development of a biofilm is a dynamic process that involves multiple steps, including cell-surface attachment, matrix production, and population expansion. Increasing evidence indicates that biofilm adhesion is one of the main factors contributing to biofilm-associated infections in clinics and biofouling in industrial settings. This review focuses on describing biofilm adhesion strategies among different bacteria, including *Vibrio cholerae*, *Pseudomonas aeruginosa*, and *Staphylococcus aureus*. Techniques used to characterize biofilm adhesion are also reviewed. An understanding of biofilm adhesion strategies can guide the development of novel approaches to inhibit or manipulate biofilm adhesion and growth.

## Introduction

Having lived on Earth for billions of years, bacteria thrive in hospitable environments, including rivers, soil, and vegetation, and further inhabit extreme environments, such as hot springs, the deep ocean, and even nuclear waste (Fredrickson et al., 2004; Fouke, 2016; Tarn et al., 2016). Bacteria in natural environments often survive in matrix-encased communities called biofilms (Hall-Stoodley et al., 2004; de la Fuente-Núñez et al., 2013). The biofilm matrix is made of extracellular polymeric substances (EPSs), which are a complex mixture consisting of exopolysaccharides, accessory proteins, lipids, and sometimes extracellular DNA (eDNA; Flemming et al., 2007). The EPS matrix enhances the adaptability and survival of bacteria in their natural niches, especially in harsh environments (Flemming and Wingender, 2010). Compared to their planktonic counterparts, matrix-embedded bacteria enjoy many evolutionary advantages (Dragoš and Kovács, 2017). For example, biofilm-dwelling cells are less susceptible to antibiotic treatment, harder to kill by the host immune system, and more resistant to mechanical forces once they attach firmly to surfaces (Stewart and Costerton, 2001; Lecuyer et al., 2011; Yan and Bassler, 2019).

The study of bacterial biofilms is highly relevant to human health. Many chronic and acute diseases, including cystic fibrosis (Moreau-Marquis et al., 2008), tuberculosis, and even dental gingivitis (Mark Welch et al., 2016), involve biofilm formation. Additionally, biofilms can survive and grow on fomites, including medical devices and implants (Levering et al., 2014), leading to nosocomial infections and widespread community transmission (Yamamoto et al., 2010). Indeed, biofilm-mediated infections exhibit a positive correlation with the development of chronic infectious diseases (Gómez and Prince, 2007; Bjarnsholt et al., 2008). On the other hand, biofilms can also be beneficial; examples include the biofilm sludge essential for wastewater treatment (Wuertz et al., 2003), plant root biofilms for nitrogen fixation (Poole, 2017), and beneficial commensal biofilms in the human gut (de Vos, 2015). Therefore, gaining a comprehensive understanding of how biofilms develop and adhere to surfaces would facilitate the screening of molecules to interfere with surface adhesion, leading to new treatments for biofilm-impacted diseases and potentially to new biofilm-based, functional materials (Huang et al., 2019).

The biofilm developmental cycle begins with planktonic cells approaching and subsequently attaching to a solid substrate (Hall-Stoodley et al., 2004). Many factors, including temperature, surface chemistry, the availability of nutrients, and fluid flow, can modulate the mechanism and strength of bacterial adhesion to surfaces (Bos et al., 1999). After settling, the surface-attached cells can move along the surface, aggregate, and start to build a three-dimensional (3D) structure through proliferation and EPS production. The EPS matrix further assists bacterial adhesion to diverse surfaces *via* divergent mechanisms depending on the species. When biofilm cells face environmental challenges, such as nutrient limitation, they undergo collective dispersal and reinitiate the biofilm developmental cycle on a new favorable surface (Rumbaugh and Sauer, 2020).

Although intuitive, the concept of biofilm adhesion is surprisingly difficult to define precisely. Unlike animals, such as squid that can rely on suction, or geckos that make use of a complex patterned surface topography (Autumn et al., 2014), biofilm-dwelling bacteria only have microscopic interactions to work with. These may include the binding of specific ligands or nonspecific interactions, such as van der Waals and hydrophobic interactions (Carniello et al., 2018). Physically, we can define adhesion as the force required to separate a biofilm from the underlying substrate. The molecular mechanisms underlying biofilm adhesion, however, can be complicated and species dependent. It is also difficult to draw a clear-cut line between the adhesion of individual bacterial cells and the adhesion of the entire biofilm, which may contain additional contributions from EPS, eDNA, and adhesion proteins, to foreign surfaces. Some biofilm-forming species possess specific adhesins that only function in the biofilm context, while other species use the same molecules for adhesion of both individual cells and biofilm-dwelling cells. Interestingly, there is an inherent “avidity” effect (Erlendsson and Teilum, 2021) for biofilm adhesion: Adhesion molecules in the biofilm matrix can bind to foreign surfaces simultaneously and increase the adhesive energy collectively; meanwhile, adhesins that function for individual cells can be amplified on the scale of the entire biofilm. We therefore include both cases in the current review with a focus on adhesion molecules that are known to be relevant for biofilm formation; readers are referred to more comprehensive reviews on well-studied adhesins that function primarily for isolated cells, such as FimH in *Escherichia coli* (Le Trong et al., 2010; Juge, 2012). We start by reviewing current technologies to study biofilm adhesion and subsequently move on to various adhesion strategies adopted by several pathogenic bacteria with direct relevance to human health. We choose two Gram-negative species, *Vibrio cholerae* and *Pseudomonas aeruginosa*, and one Gram-positive species, *Staphylococcus aureus*, as illustrative examples. Finally, we propose some potential avenues for future research.

## Techniques Used to Characterize Biofilm Adhesion

**Figure 1:**
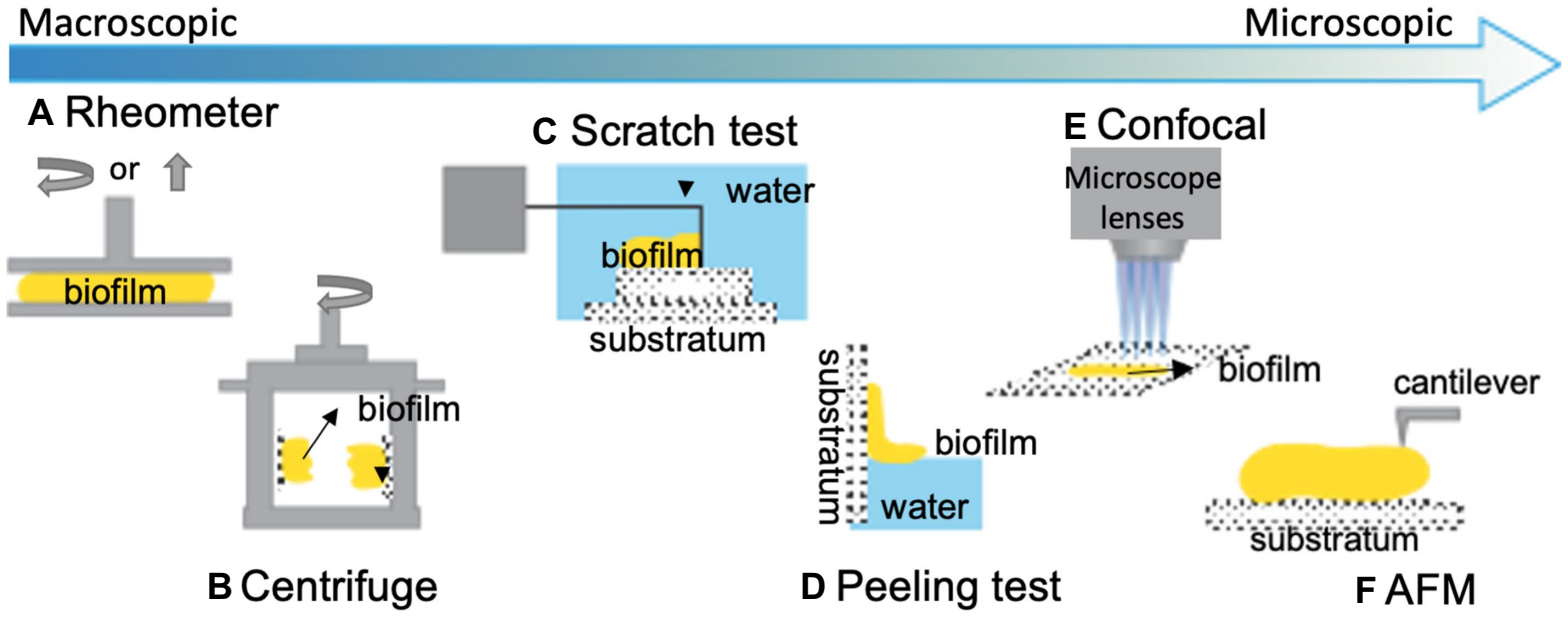
Illustration of some common techniques for quantifying biofilm adhesion. **(A)** A shear rheometer is used to determine the viscoelastic and adhesive properties of bulk biofilms with different operating modes (Gloag et al., 2018). Yellow represents biofilm. **(B)** A centrifugal force is applied to biofilm-colonized plates installed on rotary tables to evaluate the biofilm adhesive strength (Ohashi and Harada, 1994). **(C)** A T-shaped blade is used to scratch biofilms off a surface to measure the energy required to detach a biofilm from the substratum (Chen et al., 1998). **(D)** A capillary force is used to peel hydrophobic biofilms off of hydrophilic substrates and measure the strength of the biofilm-substrate interaction (Yan et al., 2018). **(E)** Additionally, confocal-based techniques and **(F)** AFM-based techniques are commonly utilized for quantifying biofilm adhesion at the cellular scale. This figure is modified with permission from Boudarel et al. (2018).

**Figure 2:**
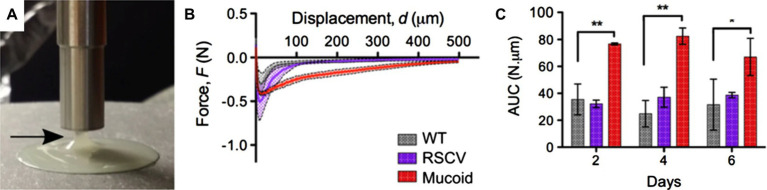
Measurement of biofilm adhesion with a rheometer. **(A)** Image of a mucoid *P. aeruginosa* colony biofilm taken as the shaft of the rheometer is pulled away from the biofilm. The arrow indicates the biofilm adhered to the probe as it is raised off the biofilm. **(B)** Force-displacement curves of 4-day-old *P. aeruginosa* colony biofilms from the unloading phase of the measurements. RSCV stands for rugose small colony variant. **(C)** Area under the curve (AUC) as a quantification of biofilm adhesion for 2-day-, 4-day-, and 6-day-old *P. aeruginosa* colony biofilms from measurements shown in **(B)**. Data presented as mean ± standard deviation (SD), *n* = 4. ^*^p < 0.01; ^**^p < 0.001. This figure is adapted with permission from Gloag et al. (2018).

A range of techniques have been established to study the adhesion of biofilms formed under both static and dynamic conditions. Biofilm adhesion can be either measured at the macroscopic or microscopic levels; Figure 1 gives a summary of existing techniques. At the macroscopic level, bulk rheology, a common technique to measure the mechanical properties of materials that possess both solid and liquid features, can be applied to quantify the viscoelasticity and adhesion of biofilms (Figure 1A). In general, biofilms can be conceptualized as porous, soft viscoelastic materials similar to hydrogels (Wloka et al., 2004); a shear rheometer is commonly employed for this purpose. When implemented in the tack test or lifting mode, a rheometer can be used to measure biofilm adhesion (Figures 2A,B). In this mode, the probe of the rheometer is lifted vertically, and the total energy needed to detach the biofilm from the probe is measured. For example, Gloag et al. adopted this method to measure the adhesive properties of biofilms formed by mucoid clinic isolates of *P. aeruginosa* (Gloag et al., 2018). By comparing pathoadapted *P. aeruginosa* variants to their isogenic wild-type (WT) parent, Gloag et al. demonstrated that mucoid colony biofilms are more cohesive compared to WT, whereas the adhesion of colony biofilms from WT and rugose small-colony variants are comparable (Figure 2C; Gloag et al., 2018). Coupling these results with other viscoelasticity measurements, they propose that biofilm mechanics should be considered as an important virulence property of biofilms. The drawback of using a rheometer for adhesion quantification is that other energy dissipation processes, such as biofilm deformation and fracturing, are involved and can even dominate during the measurement, resulting in a significant overestimation of the biofilm adhesion energy.

Another interesting macroscopic measurement is presented by Ohashi and Harada (1994; Figure 1B). They estimated the force required for biofilm detachment by imposing differential centrifugation forces on biofilms attached to a plate. The centrifugal force applied to the biofilm-attached plate can be converted into the biofilm adhesive force. However, the output data from this measurement are difficult to interpret since it is unclear whether fracture happens within the biofilm or at the biofilm-plate interface, and there are variations depending on the mode of centrifugation (Boudarel et al., 2018).

A straightforward and more popular method to measure bulk biofilm adhesion is the scratch test (Figure 1C; Chen et al., 1998). In brief, modifications are made to a micromanipulation device to include a T-shaped blade connected to a force transducer. During the scratch test, the T-shaped probe scrapes the biofilm horizontally off from the surface; the measured forces are subsequently calibrated with respect to a naïve surface. The biofilm adhesive strength can thus be defined as the work required to remove the biofilm per unit area (Chen et al., 1998). A typical value of 0.05 ~ 0.2 J/m^2^ was reported for *Pseudomonas fluorescens* biofilms, which represents one of the first quantitative measurements of biofilm adhesive strength. Later, this method was adopted by Levering et al. to measure the adhesive strength between mixed community biofilms and the inner luminal surface of urinary catheters (Levering et al., 2016). This technique provides a straightforward and repeatable measurement of biofilm adhesive strength. However, the measurement is complicated by the elastic deformation of the biofilm being scraped off of the substrate, again leading to an overestimation of the biofilm adhesive strength.

**Figure 3:**
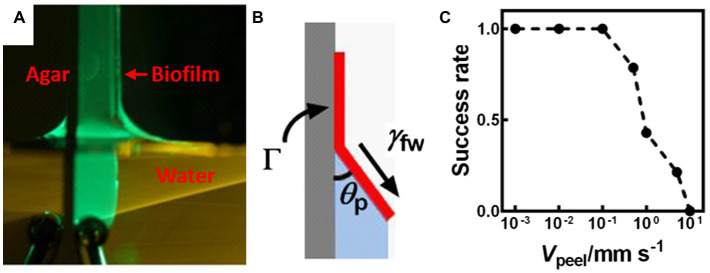
Measurement of the adhesion energy between a biofilm and a substrate using capillary peeling. **(A)** Representative image of the capillary peeling process. A *V. cholerae* colony biofilm was first grown on an agar surface. The biofilm and the agar surface were gradually dipped into water, during which the biofilm is peeled off. **(B)** Schematic representation of the capillary peeling process. The interfacial tension (*γ*_fw_) between the water and the biofilm causes peeling of the biofilm from the substrate with a constant peeling angle (*θ*_p_). Γ denotes the adhesion energy (energy/area) between the biofilm and the substrate. **(C)** The success rate of capillary peeling decreases with peeling velocity (*V*_peel_), suggesting that capillary peeling relies on equilibrium conditions rather than kinetics. This figure is reproduced with permission from Yan et al. (2018).

More recently, a capillary-peeling-based technique was developed to measure the adhesive strength of colony biofilms grown at the air-solid interface (Figure 1D; Yan et al., 2018). By slowly dipping a biofilm grown on an agar plate into water, the biofilm is peeled off by capillary forces (Figure 3A). At the water-biofilm-agar triple contact point, a characteristic angle emerges at the equilibrium condition. This angle is determined by the adhesive energy between the biofilm and the substrate (Figure 3B). Interestingly, a slower dipping velocity leads to a higher success rate for peeling (Figure 3C), confirming the quasi-equilibrium nature of the peeling process. The measured value of ~5 mJ/m^2^ for *V. cholerae* colony biofilms is much smaller than that reported by other methods, which is believed to represent the inherent interfacial energy between these biofilms and their substrates. Moreover, the authors showed that the peeled biofilms can be transferred *intact* onto another substrate, therefore enabling high-resolution imaging and potentially other biofilm-related technologies. This method is unfortunately not applicable to hydrophilic biofilms, which do not peel off.

At the microscale level, there are two types of adhesion measurements: measurements that count the amount of surface-attached biomass and measurements that determine critical forces needed for cell detachment. The former technique relies heavily on imaging and is performed by a fluorescent microscope [most commonly a scanning confocal laser microscope (SCLM; Figure 1E)]. By quantifying and comparing the total biomass on the surface before and after some perturbation, a qualitative measure of adhesive strength can be obtained. This procedure is straightforward and allows a comparison between different strains or mutants, but it is qualitative in nature and therefore commonly used in combination with critical force measurements.

The method of critical force measurement allows a quantitative understanding of the adhesive strength of microbial communities. One commonly adopted technique is atomic force microscopy (AFM; Figure 1F) or single-cell force spectroscopy (SCFS; Binnig et al., 1986; Dufrene, 2002; Helenius et al., 2008). In AFM measurements, a sharp tip or cantilever scans over a surface (Gerber and Lang, 2006). At a particular location, a force-distance curve can be generated as the cantilever approaches a cell. The cantilever can be modified with different chemicals to represent different surfaces to which cells attach. As a result, a force-distance curve can be generated that carries quantitative information on the cell-to-substratum interaction (Fang et al., 2000). AFM can provide both a high-resolution image and quantitative force measurements, and therefore, it is a powerful tool for investigating the biofilm adhesion mechanism, especially when combined with mutagenesis, biochemistry, and single-cell visualization. Having reviewed the methodology involved in measuring biofilm adhesion, we now examine several model organisms to illustrate various strategies used by bacterial biofilms to attach to surfaces.

## Gram-Negative Species – *Vibrio Cholerae*

*Vibrio cholerae* is the causative agent of pandemic cholera (Nelson et al., 2009; Charles and Ryan, 2011). *V. cholerae* cells often attach to a surface in the aquatic environment and in host intestines, which suggests that surface adhesion is an important strategy for colonization during infections and is essential for survival in natural niches (Taylor et al., 1987; Huq et al., 1996; Kirn et al., 2005; Stauder et al., 2010; Purdy and Watnick, 2011). Biofilm formation has been suggested to enhance the survival of *V. cholerae* in the aquatic ecosystem and provides protection against the acidic stomach environment in the human host (Miller et al., 1984; Alam et al., 2007; Huq et al., 2008; Tamayo et al., 2010). For example, studies show that the removal of particles larger than 20 μm from water could effectively reduce cholera incidence by 48%, suggesting that biofilms or cell aggregates contribute significantly to cholera outbreaks (Huq et al., 1996; Colwell et al., 2003).

**Figure 4:**
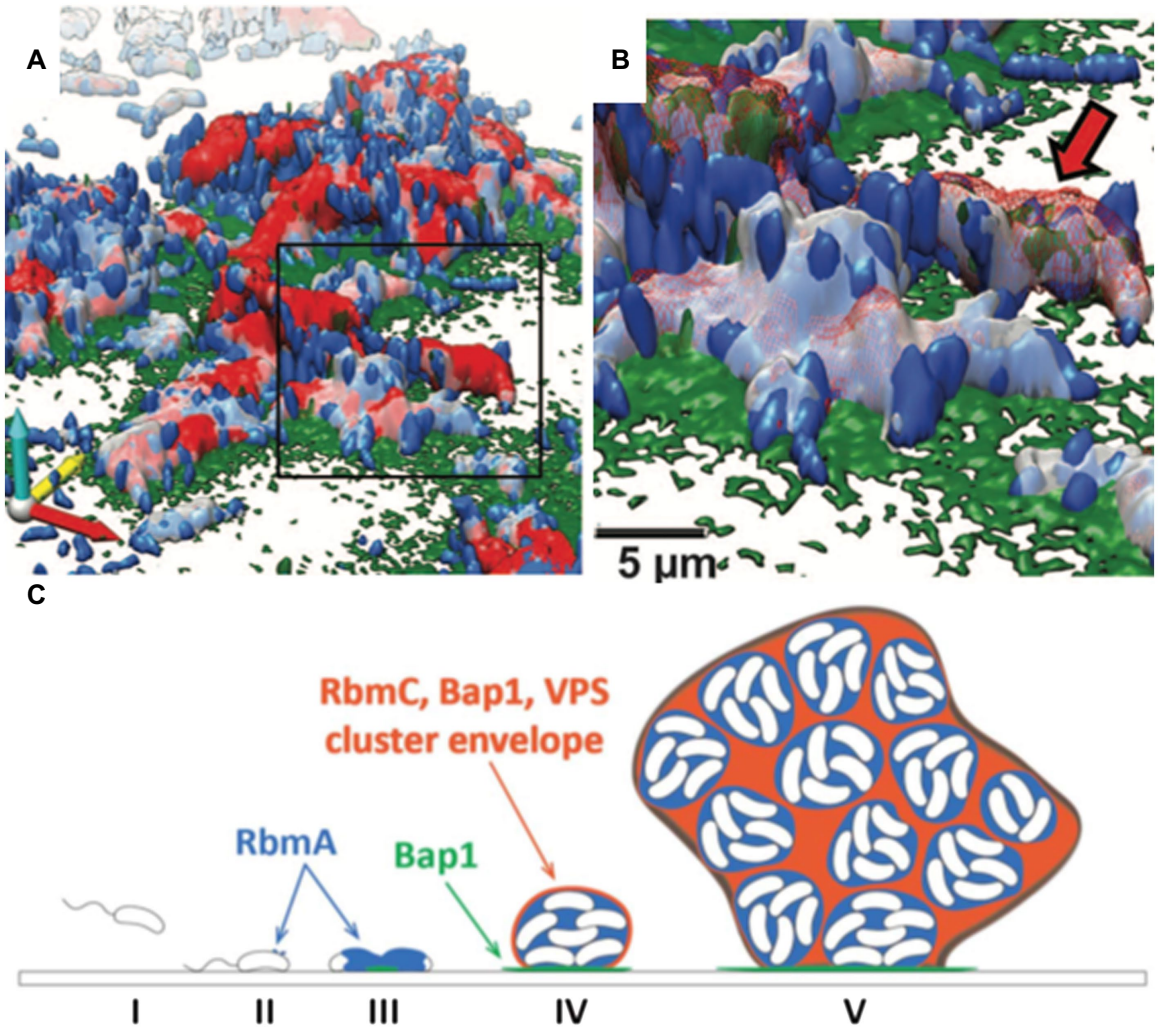
The *V. cholerae* biofilm structure. **(A)** Three-dimensional architecture of a *V. cholerae* biofilm obtained through high-resolution SCLM. Images are pseudocolored in blue (cells), gray (RbmA), red (RbmC), and green (Bap1). RbmA localizes around and within the cell cluster, whereas Bap1 and RbmC encase the cell clusters. The Bap1 signal is also concentrated at the biofilm-substratum interface. **(B)** Enlargement of the box region in **(A)**. The red arrow indicates one cell cluster. **(C)** Proposed model of biofilm development in *V. cholerae*. This figure is adapted with permission from Berk et al. (2012).

The structural integrity of biofilms is highly dependent on the production of the biofilm matrix components (Flemming and Wingender, 2010). Upon the initial attachment mediated by mannose-sensitive hemagglutinin type IV pili and flagellum, *V. cholerae* cells show a robust ability to adhere to both biotic and abiotic surfaces (Watnick et al., 1999; Fong and Yildiz, 2007). In addition, chitin-regulated pili facilitate attachment to the chitinous exoskeleton of zooplankton (Meibom et al., 2004; Reguera and Kolter, 2005). Subsequently, *V. cholerae* develops 3D biofilm structures by producing EPS matrices and *via* cell proliferation. Multiple components of the *V. cholerae* biofilm EPS have been identified, including the key polysaccharide, *Vibrio* polysaccharide (VPS), and three matrix proteins, RbmA, Bap1, and RbmC (Figure 4; Teschler et al., 2015).

*Vibrio cholerae* cells can develop into phenotypically different colonies, i.e., rugose and smooth colony variants, in response to environmental stress (Morris et al., 1996; Wai et al., 1998; Yildiz and Schoolnik, 1999). A number of studies suggest that the rugose phenotypes are associated with an increase in VPS production. The synthesis and secretion of VPS is carried out by the products of the *vps*-I and *vps*-II gene clusters (Fong et al., 2010). Deleting either the *vps*-I or the *vps*-II cluster results in a smooth colony phenotype with no VPS production. The chemical nature of VPS has been recently determined to contain glucose, galactose, and N-acetylglucosamine and is made of repeating units of an acetylated tetrasaccharide unique to *V. cholerae* (Yildiz et al., 2014; Reichhardt et al., 2015). VPS plays the dominant role in defining the biofilm structure of *V. cholerae*, and all accessory proteins depend on VPS to function (Figure 4; Fong et al., 2010).

Among the accessory proteins, much effort has focused on RbmA, which was first discovered as a secreted protein that changes the morphology of *V. cholerae* colonies on agar plates (Fong et al., 2006). Subsequently, RbmA was shown by high-resolution microscopy to adhere biofilm cells to each other (Figures 4A,B). Structural and genetics work further demonstrates that RbmA binds VPS directly and uses a binary structural switch with its fibronectin type III (FnIII) domains to modulate its function (Giglio et al., 2013; Maestre-Reyna et al., 2013; Fong et al., 2017). During the late stages of biofilm formation, *in situ* proteolysis of RbmA promotes attachment of planktonic cells to existing biofilms (Smith et al., 2015). These foundational studies have revealed the important role of RbmA in maintaining the structural integrity of *V. cholerae* biofilms.

Less is known about how *V. cholerae* biofilms adhere to surfaces. Two proteins, biofilm-associated protein 1 (Bap1) and rugosity and biofilm structure modulator C (RbmC), have been suggested to contribute to the cell-to-surface adhesion in *V. cholerae* biofilms as well as to biofilm strength (Fong and Yildiz, 2007; Teschler et al., 2015; Yan et al., 2018). While the ∆*rbmC* and ∆*bap1* single mutants possess colony morphology and adhesion capabilities similar to WT, double deletion of these genes results in floating biofilm clusters and an altered colony morphology (Absalon et al., 2011). This observation suggests that RbmC and Bap1 have partially redundant functions in *V. cholerae* biofilm adhesion. By using high-resolution SCLM, Berk et al. showed that the spatial distributions of Bap1 and RbmC are notably different at the interface between cell clusters and the substratum (Figures 4A,B; Berk et al., 2012). Bap1 appears to act as an anchor between the biofilm and the solid surface, whereas RbmC’s signal was much weaker at the biofilm-substrate interface (Berk et al., 2012; Yan et al., 2016). Both Bap1 and RbmC contribute to the formation of dynamic envelopes surrounding cell clusters, together with VPS (Figures 4C; Berk et al., 2012).

**Figure 5:**
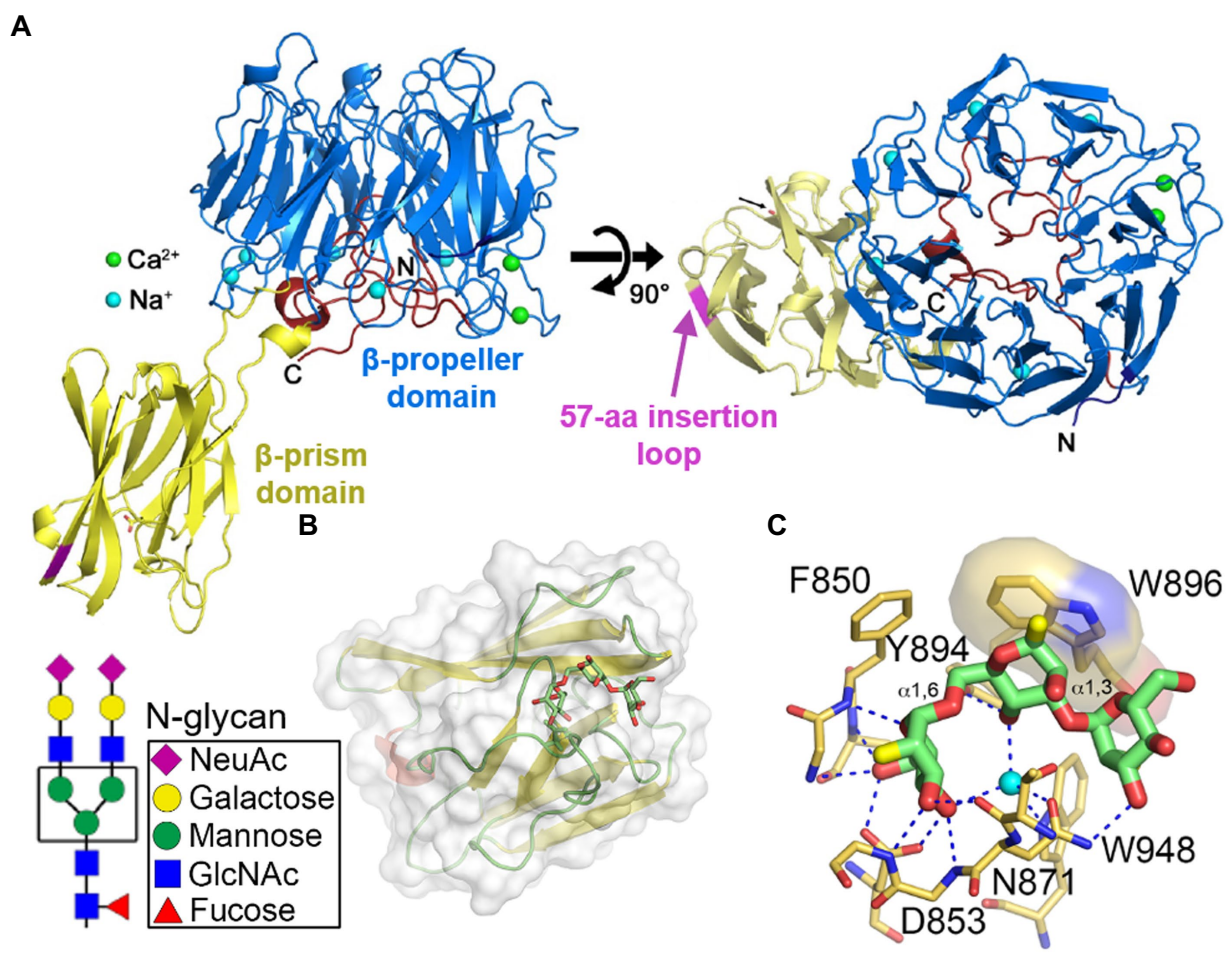
Molecular mechanism of *V. cholerae* biofilm adhesion. **(A)** Crystal structure of Bap1_Δ57_ (PDB:6MLT). The 57-amino acid insertion in the β-prism domain was removed for crystallization (indicated by magenta segment). **(B)** Crystal structure of the second β-prism domain of RbmC bound to mannotriose (PDB:5V6F). Inset shows the core N-glycan structure. **(C)** Close-up view of the binding pocket and key residues involved in glycan binding in the β-prism. Panel **(A)** is adapted with permission from Kaus et al. (2019); panels **(B,C)** are adapted with permission from De et al. (2018).

The close-to-full-length crystal structure of Bap1 has been recently solved (Figure 5A; Kaus et al., 2019). Bap1 consists of an eight-bladed β-propeller with a β-prism inserted within blade-6 *via* a flexible linker. Comparing Bap1 and RbmC, Bap1 has a 57-amino acid insertion in its β-prism domain, which renders Bap1 and GFP fusion constructs insoluble when expressed heterologously in *E. coli*. This suggested that the 57-amino acid insertion could modulate the solubility of Bap1, potentially leading to surface deposition and biofilm hydrophobicity (Berk et al., 2012; Hollenbeck et al., 2014). On the other hand, by screening against a chip-based mammalian glycan library, the β-prism domains in RbmC were found to favor complex N-glycans highly presented on mammalian cell surfaces (Marth and Grewal, 2008; Moremen et al., 2012). The crystal structures of RbmC’s β-prisms bound to segments of N-glycans confirm the screening results (Figures 5B,C; De et al., 2018) and strongly suggest that RbmC plays a role in *V. cholerae* biofilm adhesion to host intestinal surfaces. Interestingly, the binding motif between RbmC’s β-prisms and N-glycan fragments is similar to that of the *Vibrio cholerae* cytolysin (VCC) pore-forming toxin, which is known to recognize host cell surfaces using its β-prism domain (De and Olson, 2011). As a result, even though Bap1 and RbmC share similar features and have overlapping functions (Fong and Yildiz, 2007; Absalon et al., 2011; Yan et al., 2016; Kaus et al., 2019), structural differences in their β-prisms and domain organization may potentially contribute differentially to *V. cholerae* habitation on various surfaces. However, the biophysical mechanism underlying the adhesion provided by RbmC and Bap1 to *V. cholerae* biofilms is still elusive.

Besides these key factors mentioned above, there are additional matrix proteins that contribute to *V. cholerae* biofilm development. Many of those factors are encoded in the *vps* intergenic region, downstream of *rbmA* (Fong and Yildiz, 2007). Fong et al. demonstrated that in addition to *rbmA* and *rbmC*, *rbmB*, *rbmD*, *rbmE*, and *rbmF* all encode proteins that modulate *V. cholerae* rugose colony development and biofilm formation (Fong and Yildiz, 2007). Among these genes, RbmB is suggested to function as a polysaccharide lyase since the ∆*rbmB* mutant developed a more wrinkled colony biofilm with increased VPS accumulation, and the ∆*rbmB* biofilm is defective in dispersal (Singh et al., 2017; Yan et al., 2017). More recently, RbmD is suggested to contribute to biofilm formation by glycosylating other matrix proteins (Vorkapic et al., 2019), but the mechanism is still unclear. Clearly, a more comprehensive understanding of the *V. cholerae* biofilm matrix proteins and how they interact are needed.

**Figure 6:**
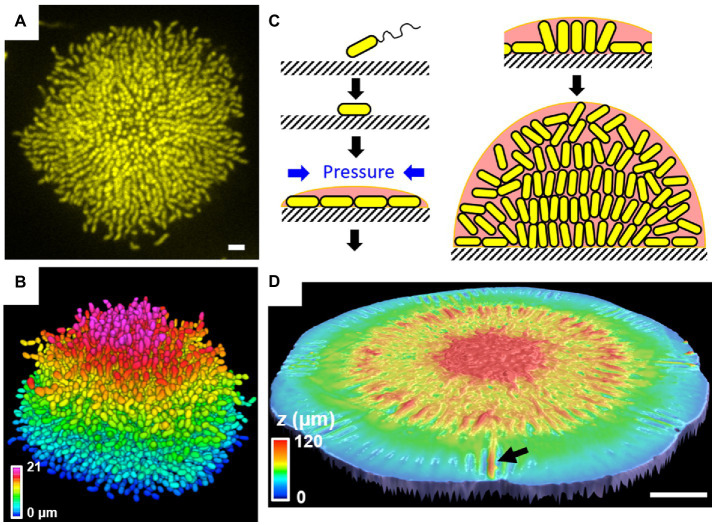
Single-cell live imaging of *V. cholerae* biofilms. **(A)** Cross-sectional image of the bottom cell layer of a growing *V. cholerae* biofilm cluster at 18 h and **(B)** the corresponding segmented image with color coding according to the z position. Scale bar: 3 μm. **(C)** Schematic model of the *V. cholerae* biofilm growth process. **(D)** Surface topography of a *V. cholerae* colony biofilm grown on 0.5% agar at the onset of the wrinkling-to-delamination transition (36 h). The arrow indicates a delaminated blister. Scale bar: 2 mm. Panels (**A**–**C**) are adapted with permission from Yan et al. (2016); panel **(D)** is adapted with permission from Yan et al. (2019).

Recent progress in single-cell resolution imaging reveals the important role of biofilm adhesion in shaping the architecture and cell ordering of *V. cholerae* biofilms. Initially, images of fixed *V. cholerae* cells obtained at different times during biofilm formation were acquired to learn how cell arrangements change as biofilms mature (Drescher et al., 2016). The community transitions from a two-dimensional (2D) branched morphology to a dense mature 3D cluster, in which vertical cells reside at the biofilm center and radially orientated cells are at the periphery. This entire sequence of structural transitions was subsequently visualized in living, growing *V. cholerae* biofilms (Figures 6A,B; Yan et al., 2016). Mutagenesis coupled with matrix labeling showed that *V. cholerae* biofilms lacking cell-to-surface adhesion due to deletion of RbmC and Bap1 exhibit normal cell density but no cell ordering, pointing to the importance of cell-to-surface adhesion in controlling cell ordering.

To further explore the forces driving structural transitions in *V. cholerae* biofilms, agent-based simulations were developed to specifically address the effect of cell-to-surface interactions (Beroz et al., 2018). When a biofilm begins to form on a surface, it expands outward from the founder cell as a thin film (Figure 6C). During expansion, cells experience increasing mechanical pressure as they divide and push against their neighbors. These neighboring cells, in turn, resist the pushing force *via* surface adhesion. Subsequently, the pressure from these opposing forces exceeds the cell-to-surface adhesive force and causes individual cells at the center of the biofilm to reorient: They transition from aligning in parallel to aligning perpendicularly to the substrate. When verticalized cells divide, their offspring projects into the third dimension, enabling the biofilm to gradually transition from a 2D surface layer to a mature 3D community. How biofilm adhesion subsequently controls the radial organization of peripheral cells is still unclear.

The effect of surface adhesion on the rugose colony morphology has also been quantitatively investigated. As previously mentioned, using a capillary-peeling-based technique, the adhesive energy between the rugose *V. cholerae* biofilm and the substrate is measured to be ~5 mJ/m^2^, whereas the ∆*rbmC* ∆*bap1* double mutant shows negligible adhesion in this assay (Yan et al., 2018). Mechanical measurements and modeling suggest that such wrinkled morphologies arise from a macroscopic mechanical instability (Yan et al., 2019). Specifically, it was shown that the mismatch between the growing biofilm layer and the non-growing substrate causes mechanical instabilities that enable the biofilm to transition from a flat to a wrinkled film and subsequently to a partially detached film containing delaminated blisters (Figure 6D). The mechanical compression arises from surface friction when a colony biofilm expands on the agar plate, as shown by a subsequent modeling study (Fei et al., 2020). RbmC and Bap1 have been shown to play a critical role in determining the colony morphology: When they are absent, delamination occurs easily and the delaminated blisters collapse onto each other, while the blisters in wild-type rugose colonies are homogeneously distributed throughout the colony circumference (Yan et al., 2019). While the wrinkle-to-delamination model provides the conceptual guidance to understand the rugose colony morphology, many of the morphological features remain to be explained by quantitative theories.

## Gram-Negative Species – *Pseudomonas Aeruginosa*

*Pseudomonas aeruginosa* is a Gram-negative species with a large genome (6.3 million base pairs) and numerous regulatory two-component systems and transcriptional regulators, making it remarkably capable of adapting to different environments (Rodrigue et al., 2000). Biofilm formation in *P. aeruginosa* has been studied intensively due to its immediate clinical relevance: *P. aeruginosa* biofilms are commonly found in the defective mucus layer in the lungs of cystic fibrosis patients (Lyczak et al., 2002; Moreau-Marquis et al., 2008), as well as in chronic and burn wounds (Gjødsbøl et al., 2006; Brandenburg et al., 2019). The spatial organization of *P. aeruginosa* biofilms was first visualized by SCLM and contributes much to our current understanding of biofilm architecture in general (Lawrence et al., 1991; Reichhardt and Parsek, 2019). SCLM images show that *P. aeruginosa* biofilms have a 3D complex, hydrated structure with rod-shaped cells embedded in a matrix permeated with water channels. However, *P. aeruginosa* can also form different biofilm phenotypes depending on the strain and the growth conditions. For example, *P. aeruginosa* biofilms assume a characteristic “mushroom” shape when growing in flow chambers supplied with glucose as the carbon source. In contrast, *P. aeruginosa* forms flat, uniform, and densely packed biofilms when growing with citrate as the major carbon source (Klausen et al., 2003).

*P. aeruginosa* biofilm development has been characterized to involve five stages: reversible attachment, irreversible attachment, maturation-1, maturation-2, and dispersion (Sauer et al., 2002). In this review, we will primarily focus on the adhesive aspects of *P. aeruginosa* biofilms and the reversible and irreversible attachment stages; biofilm development and its regulation in *P. aeruginosa* have been extensively reviewed elsewhere (O’Toole and Wong, 2016; Rumbaugh and Sauer, 2020; Thi et al., 2020). In the reversible attachment stage, planktonic *P. aeruginosa* cells use their polar flagella to swim toward a substrate and temporarily adhere *via* their cell poles (O’Toole and Kolter, 1998; Schniederberend et al., 2019). At this stage, attachment is still reversible, and adhesion is achieved through weak, reversible interactions, including van der Waals forces. Once attached, *P. aeruginosa* cells twitch by extending and retracting their type IV pili on a surface (Chiang and Burrows, 2003; Limoli et al., 2019). Type IV pili are hair-like protein polymers that enable adhesion to host cells and surfaces (Burrows, 2012). The type IV pili are also crucial for mediating irreversible cell-to-surface attachment, colonization, DNA uptake, and virulence induction (Saiman et al., 1990; Zoutman et al., 1991).

**Figure 7:**
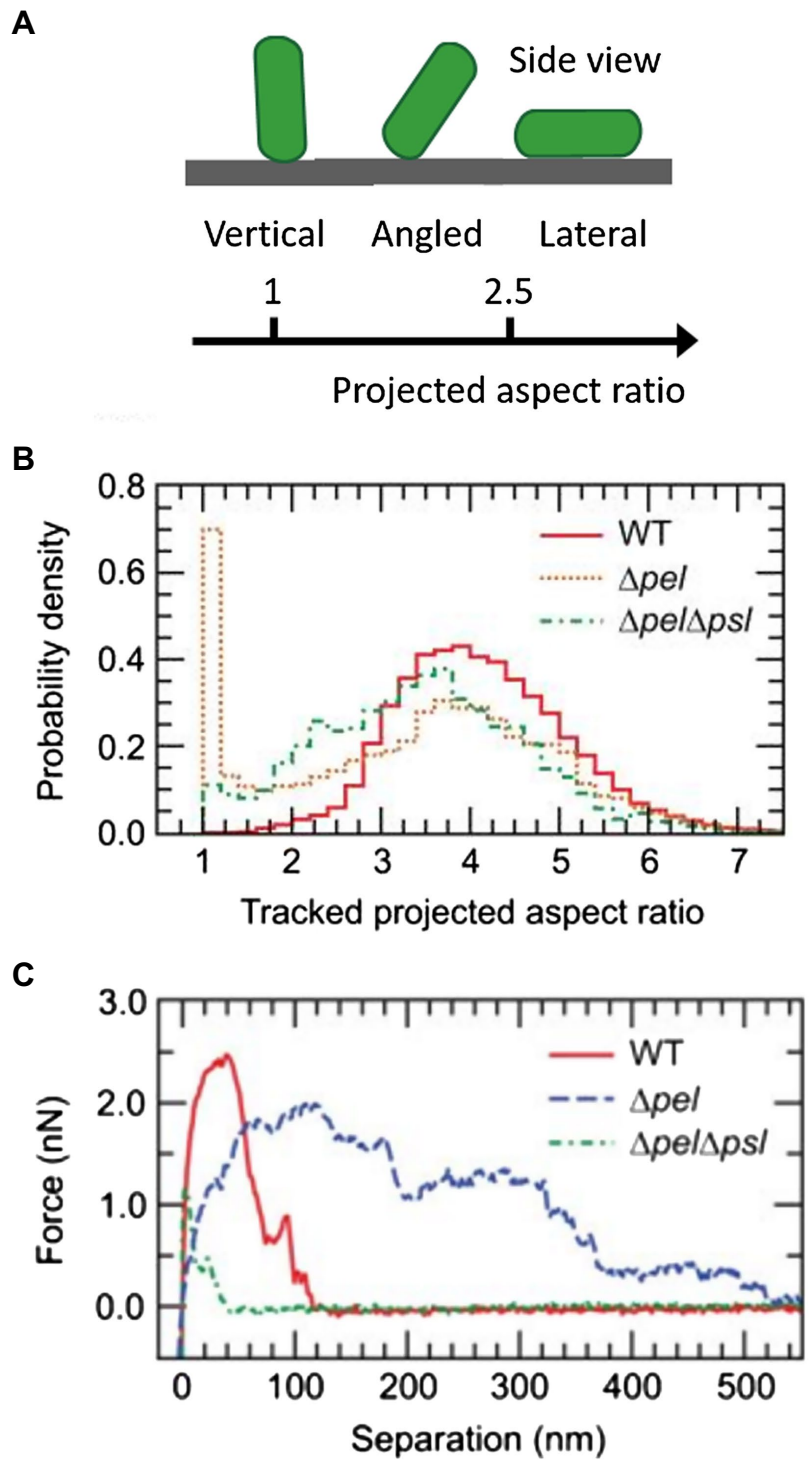
Analysis of the initial attachment phase of *P. aeruginosa*. **(A)** Schematic representation of the cell aspect ratio in a 2D projection. **(B)** Normalized histograms of the projected aspect ratios of WT, Δ*psl*, and Δ*pel*Δ*psl P. aeruginosa* cells. The position of the main peak for the Δ*pel*Δ*psl* strain is shifted to the left of the main peak for WT and Δ*psl* strains, indicating that the Δ*pel*Δ*psl* cells spend more time in a configuration tilted from the surface. For Δ*psl* cells, a second peak near 1 appears, corresponding to bacteria standing vertically on the substrate. **(C)** Representative individual force curves, showing adhesive characteristics of each strain. Δ*pel*Δ*psl* has an adhesion range significantly shorter than that of WT. Δ*pel* has a longer range of adhesion, but the magnitude is smaller than that of WT. Panels **(B,C)** are adapted with permission from Cooley et al. (2013).

In stage II, *P. aeruginosa* cells initiate irreversible attachment by generating strong adhesive forces and aligning their long axes parallel to the surface (O’Toole and Kolter, 1998). The local cell density increases through population growth, setting the stage for subsequent biofilm formation (Ha and O’Toole, 2015). In the context of infection, the type IV pilus is also believed to initiate the adhesion between *P. aeruginosa* cells and the host surface (Laventie et al., 2019). For instance, studies have shown that the type IV pili bind directly to the glycolipids asialo-GM1 and asialo-GM2 on epithelial cell surfaces (Comolli et al., 1999). The type IV pilus protein, PilY1, is a pilus-associated adhesin that has been proposed to be required for type IV pili biogenesis and attachment of *P. aeruginosa* to human tissue (Heiniger et al., 2010; Kuchma et al., 2010; Johnson et al., 2011). Luo et al. compared the aspect ratio (Figure 7A) of 2D projections of the *pilY1* mutant with that of WT cells (Luo et al., 2015). In the reversible attachment stage, both the *pilY1* mutant and WT cells vertically adhere to the surface with a projected aspect ratio around 1. However, in the irreversible attachment stage, the *pilY1* mutant has an average projected aspect ratio significantly smaller than the laterally adhered WT cells, indicating weaker adhesion. They further showed that PilY1 regulates cell-to-surface attachment *via* two separate mechanisms: (i) The Pil-Chp complex senses external signals to induce cAMP production, and cAMP, in turn, activates the transcription of *pilY1* gene and (ii) the PilY1 located on the outer membrane can induce activity of the diguanylate cyclase SadC by signaling through type IV pili. SadC has a GGDEF domain that catalyzes c-di-GMP production and therefore promotes biofilm formation (Simm et al., 2004; O’Toole and Wong, 2016).

After the initial attachment stages, *P. aeruginosa* cells aggregate to form clusters by producing EPS, which generates a more potent adhesive force allowing cell clusters to irreversibly attach to the surface. EPS also promotes biofilm mechanical strength by enhancing cell-to-cell cohesion. In *P. aeruginosa*, EPS consists of several polysaccharides, proteins, nucleic acids, and lipids. *P. aeruginosa* employs three major exopolysaccharides: alginate, Psl (polysaccharide synthesis locus), and Pel (pellicle polysaccharide; Ghafoor et al., 2011). Alginate is a linear polysaccharide composed of D-mannuronic acid and L-guluronic acid, which is the main structural component in mucoid *P. aeruginosa* biofilms. Biofilms formed by non-mucoid *P. aeruginosa* strains primarily use Pel and Psl exopolysaccharides (Colvin et al., 2012). Several studies have shown that Psl is mainly involved in the initial attachment of cells and early biofilm formation, whereas Pel is essential for late-stage biofilm formation and maturation (Friedman and Kolter, 2004; Jackson et al., 2004; Overhage et al., 2005; Campisano et al., 2006; Ghafoor et al., 2011). Psl is a mannose-rich, branched polysaccharide containing D-mannose, D-glucose, and L-rhamnose (Byrd et al., 2009), and it acts as a “molecular glue” to help *P. aeruginosa* attach to biotic and abiotic surfaces and initiate biofilm formation (Friedman and Kolter, 2004; Ma et al., 2006; Byrd et al., 2010; Zhao et al., 2013; Jones and Wozniak, 2017). In a mature biofilm, Psl contributes to the biofilm mushroom cap by forming a peripheral meshwork covering the cap region (Ma et al., 2006, 2009). Pel has recently been characterized as a N-acetyl glucosamine- and N-acetyl galactosamine-rich polysaccharide (Jennings et al., 2015). It has been shown to play a critical role in pellicle formation at the air-liquid interface, and it interacts with eDNA in the matrix through electrostatic interactions (Jennings et al., 2021). In the laboratory strain PAO1, Psl has been shown to contribute significantly to the formation of characteristic mushroom-shaped biofilms, whereas Pel contributes mainly to the biofilm cell density and/or compactness and remodeling in the late stage (Ghafoor et al., 2011; Chew et al., 2015). In the PA14 strain, however, Pel is the dominant extracellular polysaccharide and is essential for the wrinkled colony phenotype observed on agar plates (Friedman and Kolter, 2004). It will be interesting, in the future, to apply the above wrinkle-to-delamination model to PA14 colony biofilms to test the generality of the model to other systems.

**Figure 8:**
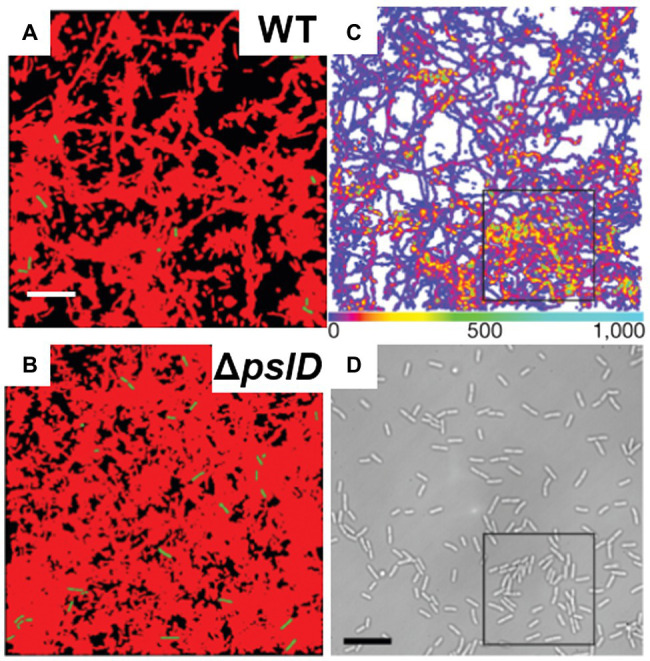
*P. aeruginosa* adhesion controls surface motility and colonization. **(A)** and **(B)** illustrate the efficiency of surface coverage by bacterial trajectories with Psl trails. Shown are cumulative surface coverage images at 5 h for WT **(A)** and Δ*pslD* **(B)**, respectively. Red corresponds to areas visited by *P. aeruginosa*, and black corresponds to the unvisited areas. **(C)** Visit frequency map of WT cells for the first 15.7 h after inoculation, when microcolonies are just starting to form. The black square indicates an example of colony formation. **(D)** The brightfield image corresponding to **(C)**. Scale bars, 10 μm. This figure is reproduced with permission from Zhao et al. (2013).

Studies focused on the biophysical and biomechanical mechanisms of how *P. aeruginosa* cells attach to surfaces and self-organize into biofilms have revealed Psl as a key component of adhesion. For instance, Zhao et al. used cell-tracking algorithms in combination with Psl staining to map the spatial characteristics of cell-to-surface interactions for WT *P. aeruginosa* as well as for the Δ*pslD* mutant deficient in Psl production (Zhao et al., 2013). Their study shows that the surface exploration patterns of WT and Δ*pslD P. aeruginosa* are dramatically different (Figures 8A,B). WT *P. aeruginosa* cells migrate across a substratum leaving Psl footprints, which in turn enhance subsequent cell adhesion and eventual promotion of microcolony formation (Figures 8C,D). They dubbed this phenomenon “the rich get richer.” The Δ*pslD* mutant does not display this phenomenon. To explain this difference, they proposed that type IV pili favor Psl-rich regions pulling cells toward these regions. Later, Cooley et al. used optical microscopy in combination with AFM to examine the role of these polysaccharides in promoting cell-to-surface adhesion (Figures 7B,C; Cooley et al., 2013). Their result demonstrates that Pel alone can provide some transient cell-to-surface attachment, but not as permanent as Psl. Furthermore, force measurements showed that Psl generates short-ranged and localized adhesive forces, whereas Pel generates weaker but longer-ranged adhesive forces. By comparing the projected aspect ratio of Δ*psl*, Δ*pel*, and WT cells, the authors reached the conclusion that Pel controls the attachment geometry by helping the rod-shaped *P. aeruginosa* cells lie parallel to the substrate. Therefore, the Δ*pel* strain can still permanently adhere to a surface, but with only one end attached, whereas the WT cells can lie parallel to surface with little to no tilting. How Psl and Pel interact at the molecular level to ensure the parallel alignment remains to be demonstrated.

In addition to the critical role of exopolysaccharides, eDNA makes an important contribution to *P. aeruginosa* biofilm formation. eDNA binds to Ca^2+^ forming “cationic bridging” and therefore overcomes the electrostatic repulsion between negatively charged polysaccharide strands (Bos et al., 1999; Whitchurch et al., 2002; Flemming et al., 2007; Das et al., 2014; Powell et al., 2018). The cationic bridge also helps initial adhesion to foreign surfaces, facilitates cell aggregation, and strengthens *P. aeruginosa* biofilms. Furthermore, eDNA can interact with Pel and Psl to form fiber-like networks that likely promote stability of the biofilm (Jennings et al., 2015; Wang et al., 2015; Jennings et al., 2021).

Finally, accessory proteins also exist in the *P. aeruginosa* biofilm matrix, but their roles are not well understood at this time. The best characterized matrix protein is CdrA. CdrA is an extracellular adhesin that is found in both cell-associated and secreted forms in biofilms. CdrA is secreted out of the cell as cargo of the CdrA-CdrB two-partner secretion system (Reichhardt et al., 2018). Full-length CdrA is a 220 kDa protein that is processed by a poorly understood proteolysis mechanism to generate a 150 kDa truncated released form of the protein. Recent studies indicate that the C-terminus of CdrA is cleaved by the periplasmic protease LapG (Rybtke et al., 2015; Cooley et al., 2016). LapG is regulated by the intracellular levels of c-di-GMP such that under conditions of low c-di-GMP, LapG cleaves cell-associated CdrA at a C-terminal TAAG site to release CdrA into the environment. CdrA contributes to the maintenance of the structural integrity of *P. aeruginosa* aggregates *via* CdrA-Psl, CdrA-Pel, and CdrA-CdrA interactions (Borlee et al., 2010; Reichhardt et al., 2018, 2020). Interestingly, CdrA can also promote cell clustering without EPS (Reichhardt et al., 2018); the exact mechanism is unclear.

In addition to CdrA, *P. aeruginosa* also produces two small soluble lectins, LecA and LecB, that bind to the repeating sugar unit in the exopolysaccharide and help *P. aeruginosa* adhere to targets in the host organism (Mitchell et al., 2005; Visini et al., 2015; Passos da Silva et al., 2019). In particular, LecA is a tetrameric protein that binds to sugars, such as N-acetyl-D-galactosamine, galactose, and glucose. Although each 12.8 kDa LecA monomer has a calcium-dependent binding site for galactose and an independent binding site for glucose, it is unclear if LecA binds to Psl, Pel, or both (Mitchell et al., 2002; Blanchard et al., 2014). In contrast, LecB binds to mannose residues along the backbone of Psl to promote the retention of Psl within the biofilm matrix (Passos da Silva et al., 2019). LecB is a tetrameric protein comprised of four 11.7 kDa subunits each containing a calcium-dependent ligand binding site (Loris et al., 2003) and is associated with the outer membrane porin OprF (Funken et al., 2012). An interesting direction for future endeavors would be to search for extracellular proteins in *P. aeruginosa* biofilms that, like RbmC and Bap1 in *V. cholerae*, serve as biofilm-specific adhesins.

## Gram-Positive Species – *Staphylococcus Aureus* and Other Staphylococci

Gram-positive pathogens produce various factors known as cell wall-associated (CWA) proteins that are attached to the thick peptidoglycan cell wall through covalent linkages. Many Gram-positive strains use CWA proteins to bind to components of the mammalian extracellular matrix (ECM), such as fibrinogen, fibronectin, and cytokeratin, which, in turn, associate with proteins found on the surface of mammalian cells (Geoghegan et al., 2009; Hymes and Klaenhammer, 2016). Due to the high affinity of binding to the mammalian ECM, *S. aureus* is one of the major Gram-positive pathogens that causes acute, chronic, and sometimes life-threatening biofilm-associated diseases in humans, including sepsis, endocarditis, osteomyelitis, and pneumonia. *S. aureus* has the capacity to attach to both biotic and abiotic surfaces, resulting in a high probability of being introduced during the implantation of medical devices (Heilmann, 2011).

Attachment of staphylococci to abiotic surfaces is thought to be facilitated by various physical forces, such as hydrogen-bonding, ionic, and hydrophobic interactions. For example, AFM measurements have shown that on hydrophobic surfaces, adhesion of the hydrophobic staphylococcal cells is instantaneous, while on hydrophilic glass, gradual strengthening of the interaction is observed, which is attributed to hydrogen bond formation (Boks et al., 2008). The interaction range can be as long as 50 nm, suggesting that large surface-associated structures are responsible for adhesion (Thewes et al., 2014). *In vitro*, staphylococci can form biofilms on abiotic surfaces, but biofilm adhesion and formation are significantly enhanced if the surfaces are treated with ECM-containing media, or simply fibronectin (Chen et al., 2012; Kim et al., 2014). *In vivo*, direct contact between staphylococcal cells and abiotic surfaces is considered not relevant because the inserted abiotic surfaces are covered with ECM, with fibronectin being the dominant factor (Francois et al., 2000; Otto, 2008). Therefore, below we focus on the proteins responsible for the staphylococci-ECM interaction.

The most well-studied CWA proteins belong to the family of microbial surface component recognizing adhesive matrix molecules (MSCRAMMs). MSCRAMMs include a wide variety of adhesins, many of which have the ability to bind multiple ligands resulting in some degree of crosstalk between adhesins and target ligands. MSCRAMMs are present abundantly in many Gram-positive species, such as *Staphylococcus pseudintermedius* and *Enterococci faecalis* (Liu et al., 2007; Ponnuraj and Narayana, 2007; Bannoehr et al., 2011). *S. aureus* has about 20 MSCRAMMs, many with multiple target ligands that can bind to different surfaces in different environments (Foster et al., 2014). A common feature of *S. aureus* adhesins within the MSCRAMM protein family is the tandemly-linked IgG-like folds (Foster et al., 2014). The defining feature of CWAs is a conserved LPXTG motif at their C-terminus, which is involved in anchoring these proteins to bacterial peptidoglycans in the cell envelope (Mazmanian et al., 1999; Clarke and Foster, 2006).

**Figure 9:**
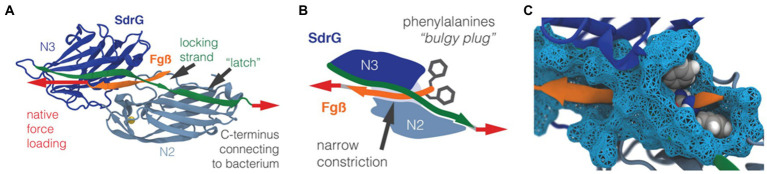
Structure of the SdrG-Fgβ complex. **(A)** The crystal structure of the SdrG (blue): Fgβ (orange) complex. The locking strand (green) encloses the peptide in the binding pocket between the Ig-fold N2 (light blue) and N3 (dark blue) domains and a calcium (yellow) binding loop. The red arrows indicate the force applied to the molecular complex. **(B)** Schematic of the “bulgy plug” hypothesis. The bulky phenylalanine side chains (gray) of Fgβ (orange) are blocked by the locking strand (green). **(C)** Crystal structure showing the bulky phenylalanine side chains in van der Waals representation (gray spheres) of Fgβ (orange), which have to wiggle through a narrow constriction (cyan surface). This figure is adapted with permission from Milles et al. (2018).

The versatile nature of MSCRAMM adhesins allows *S. aureus* to attach to a wide range of surfaces of both biotic and abiotic origin. On biotic surfaces, the cell-to-surface interactions are well-defined through specific ligand binding. For example, collagen-binding adhesin, fibronectin-binding proteins, and fibrinogen-binding proteins are some MSCRAMMs that enable *S. aureus* to attach to the mammalian ECM. Ponnuraj et al. proposed a binding mechanism called “dock, lock, and latch (DLL)” that describes the mode of action for a fibrinogen-binding MSCRAMM protein in *S. epidermidis* called SdrG (Ponnuraj et al., 2003). The crystal structure (Figure 9A) of the fibrinogen binding domain in SdrG has a similar structure to the Sdr family protein SdrE in *S. aureus* discovered later (Ponnuraj et al., 2003; Luo et al., 2017). Well-defined electron density for most of the residues of the fibrinogen-derived peptide is observed in the crystal structure, indicating that the peptide fits tightly into the cleft of SdrG’s binding domain. The DLL mechanism describes a multiple-step adhesion process: First, the C-terminal domain of SdrG forms a cover, which allows it to “lock” on to a “docked” peptide and subsequently sequester the “docked” peptide by “latching” onto the neighboring N2 domain (Figures 9B,C; Milles et al., 2018). Due to the high structural similarity of the proteins in the MSCRAMM family, it has been suggested that this DLL mechanism could be generalized to other structurally similar CWA proteins, such as FnBPA and SdrE (Ponnuraj et al., 2003; O’Neill et al., 2008; Zhang et al., 2017).

**Figure 10:**
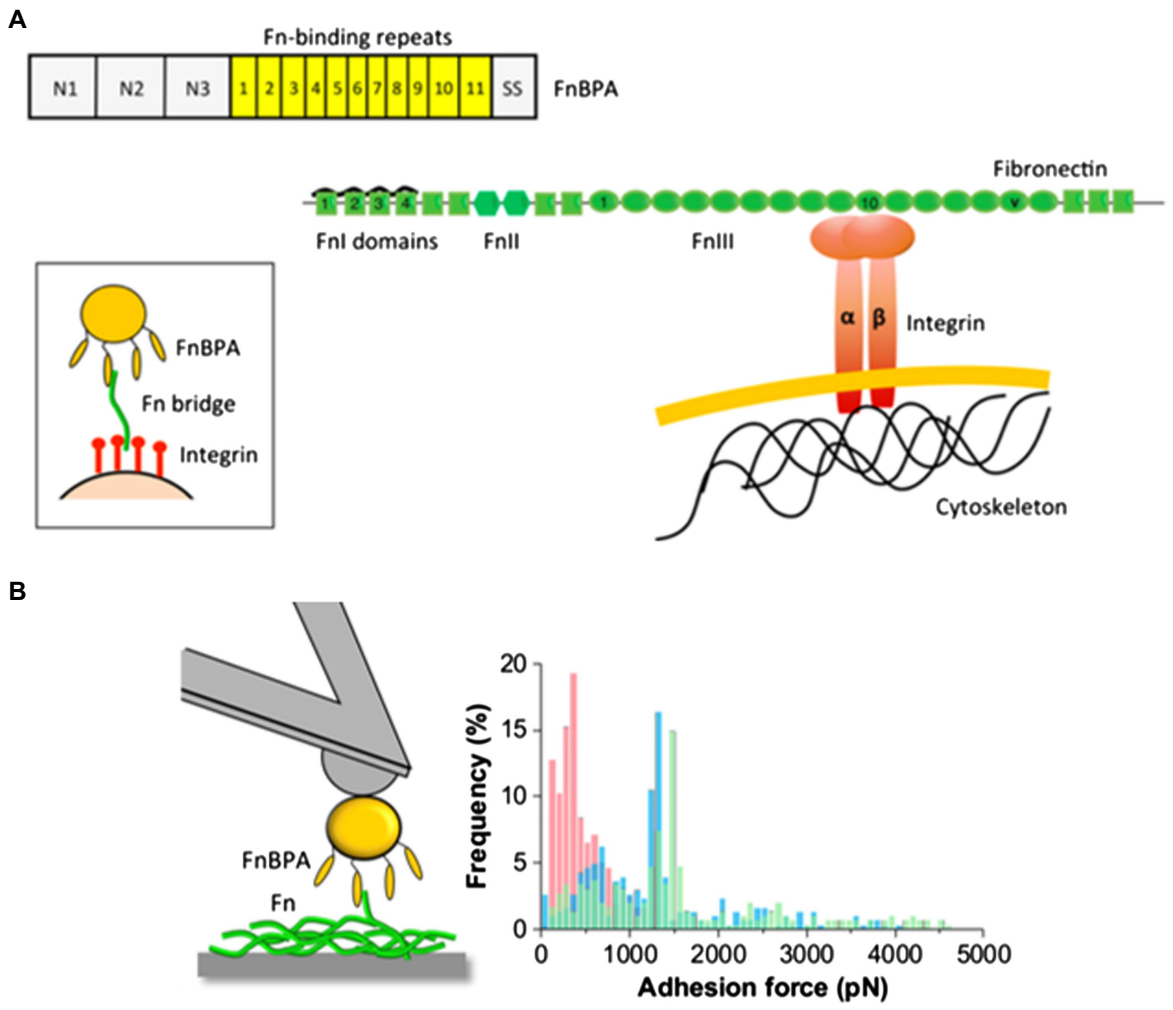
The three-component FnBPA-fibronectin-(Fn)-integrin system. **(A)** Mechanism of FnBP-dependent cell invasion by *S. aureus*. The main invasion pathway of *S. aureus* involves interaction of the Fn-binding repeats of FnBPA with type I Fn modules *via* a tandem β-zipper structure. This triggers a conformational change in Fn, resulting in the exposure of the cryptic integrin-binding site in the tenth FnIII module, which in turn engages in a high-affinity interaction with the α5β1 integrin found on the membrane of mammalian cells. **(B)** Maximum adhesion force histograms obtained by recording force-distance curves between *S. aureus* cells and Fn immobilized on solid substrates. This figure is adapted with permission from Prystopiuk et al. (2018).

Recently, Milles et al. performed AFM-based single-molecule force spectroscopy to show that SdrG binds to the N-terminus of the β-chain of human fibrinogen (Fg) with ultra-strong (~2 nN) forces, comparable to covalent interactions (Milles et al., 2018). More interestingly, this strong binding is independent of the side chains on Fg (Milles et al., 2018). The underlying molecular mechanism for the side-chain independence is that the DLL mechanism creates a deep and rigid binding pocket to confine the peptide target in a stable geometry, which only relies on backbone hydrogen bonds. The authors also used steered molecular dynamics simulations to corroborate the AFM measurements and showed that other adhesins from *S. aureus*, including the clumping factors A and B (ClfA and ClfB), SdrE, and FnBPA, all have such side-chain-independent mechanostability. Such side-chain-independent mechanism might confer an invasive advantage to staphylococci. Similarly, in a subsequent work, Prystopiuk et al. unraveled the molecular interactions underlying the three-component FnBPA-Fn-integrin system (Figure 10A; Prystopiuk et al., 2018). *S. aureus* adheres to endothelial cells *via* Fn bridges, established by FnBPA binding to α5β1 integrins on the host cell surface (Sinha et al., 1999; Massey et al., 2001; Sinha and Herrmann, 2005; Edwards et al., 2010). Their results demonstrated that FnBPA mediates bacterial adhesion to soluble Fn *via* strong forces (~1.5 nN) comparable to SdrG-Fgβ binding (Figure 10B), using a β-zipper mechanism (Prystopiuk et al., 2018).

Interestingly, *S. aureus* adhesion can be enhanced by shear forces (Geoghegan and Dufrene, 2018). For example, under high-shear conditions, ClfA binds to fibrinogen and promotes platelet capture, leading to thrombus formation. Over the past few years, an increasing number of studies have demonstrated that the *S. aureus* adhesins ClfA and ClfB can act as force-sensitive molecular switches to promote cell-to-surface adhesion under mechanical stress (Vitry et al., 2017; Herman-Bausier et al., 2018). Dufrene et al. showed that at low tensile force, the bond between ClfA and immobilized fibrinogen is weak (~0.1 nN; Herman-Bausier et al., 2018); however, with mechanical tension, the bonding force increases to ~1.5 nN. In a parallel study by the same group, it was found that ClfB adheres to loricrin through similar dynamic conformational changes (Vitry et al., 2017). They also demonstrated that the strength of the ClfB-loricrin interaction increases with mechanical tension. In both cases, an adhesive force of 1.5–2 nN is typically measured at high mechanical tension, which corresponds to the strength of single DLL-like bonding interactions. Together, these unique adhesion mechanisms enable *S. aureus* cells to modulate adhesion by sensing external forces at the molecular level, which provides significant advantages during colonization.

*S. aureus* also possesses adhesins that are not covalently attached to the cell wall. The major adhesins in this class are secretable expanded repertoire adhesion molecules (SERAMs), including the extracellular fibrinogen binding protein, extracellular matrix binding protein, and extracellular adherence protein (Eap, also known as Map and p70). Eap is the most well-studied protein in the SERAMs family. The protein sequence of Eap lacks the LPXTG motif, suggesting that Eap is associated but not covalently linked to the peptidoglycan cell wall. Over the past few decades, Eap has been shown to be an anchorless protein that contributes to staphylococcal adhesion and is capable of modulating the inflammatory response *via* interactions with the glycoprotein ICAM-1. While Eap has been shown to bind a number of host extracellular components, the exact mechanism of how Eap is involved in bacterial adhesion to these components is still unclear (Hammel et al., 2007; Geraci et al., 2017).

The adhesion proteins mentioned above are mostly produced and primarily important during the initial cell attachment phase. In staphylococci, the formation of a biofilm structure further requires the production of polysaccharide intercellular adhesin (PIA) and the release of eDNA from dead cells. PIA is synthesized by enzymes encoded by the *icaADBC* operon and plays a crucial role in the accumulation of biofilms (Fluckiger et al., 2005; Archer et al., 2011). Vuong et al. suggested that PIA may aid in the extracellular biofilm matrix stickiness *via* its electrostatic interaction with other surface polymers (Vuong et al., 2004). More recently, Ganesan et al. purified PIA from *Staphylococcus epidermidis* and measured its molecular mass (Ganesan et al., 2013). Using extensive rheological tools, they have quantitated the entanglement and association contribution to its polymer properties. More recently, the same group showed that the positively charged PIA molecules electrostatically interact with the negatively charged cells to form phase-separated, biofilm-like aggregates (Stewart et al., 2015). They suggested that such a phase separation mechanism underlies the mechanical properties of *S. epidermidis* biofilms (Stewart et al., 2015). An interesting mechanistic question for future exploration is the potential synergistic interaction between PIA (or other cell-cell aggregation factors) and the MSCRAMMs.

Furthermore, some staphylococci can also form PIA-independent biofilms; in these biofilms, several surface-associated proteins contribute to biofilm development and promote intercellular adhesion, such as the biofilm-associated protein (Bap; Cucarella et al., 2001; Tormo et al., 2005; Lasa and Penadés, 2006). Bap, a large protein of 2,276 amino acids, was first discovered through a transposon screen using adhesion to polystyrene surfaces as a phenotype (Cucarella et al., 2001); it is suggested to be involved in the primary adhesion to abiotic surfaces as well as in biofilm formation. Bap is anchored to the cell wall and subsequently undergoes processing to release fragments of the N-terminal domain, which self-assemble into amyloid fibers to form the biofilm scaffold (Taglialegna et al., 2016). This self-assembly process only takes place under low Ca^2+^ concentrations and acidic pH, suggesting that Bap also acts as a sensor of the extracellular environment (Taglialegna et al., 2016). So far, the *bap* gene has only been found in *S. aureus* isolates from bovine but not from human (Cucarella et al., 2004). *S. epidermidis* carries *bhp*, a homology of *bap*, but *bhp* does not seem to be involved in biofilm formation (Lasa and Penadés, 2006). On the other hand, *S. xylosus* forms Bap-dependent biofilms (Schiffer et al., 2019). Many mysteries still surround this relatively new biofilm adhesin in terms of its structure, function, distribution in staphylococci, and involvement in pathogenicity.

Another interesting class of matrix protein in staphylococci biofilms is accumulation-associated protein (Aap) in *S. epidermis* and *S. aureus* surface protein G (SasG) in *S. aureus*. Aap and SasG share 54% sequence identity; they both possess a signal peptide at the N-terminus, followed by an A domain and a B region with 3–12 repeating G5 and E domains of 128 amino acids (Conlon et al., 2014). These proteins also have a LPDTG motif at their C-terminus, suggesting that they are CWAs. Expression of SasG or Aap causes biofilm formation, and this induction is independent of PIA (Rohde et al., 2005; Corrigan et al., 2007). Functional Aap (220 kDa) requires proteolytic processing into a truncated 140 kDa fragment in which the A domain is cleaved (Hussain et al., 1997; Conlon et al., 2014). The unprocessed form of Aap with an intact A domain has been shown to promote primary surface adhesion only in some specific strains (Conlon et al., 2014). More generally, cleavage is necessary to expose the B region and promote cell-cell adhesion *via* two distinct Zn^2+^-dependent mechanisms: (1) the B region forming twisted rope-like structures between neighboring cells, catalyzed by Zn^2+^ (Conrady et al., 2013), and (2) the B-repeat self-assembling into functional amyloid fibers (Yarawsky et al., 2020). Interestingly, expression of SasG inhibits the MSCRAMM-mediated adhesion to host surfaces – a steric hindrance caused by SasG fibrils prevents short-ranged interactions with MSCRAMMs (Corrigan et al., 2007). This observation leads to the interesting question of how staphylococci deploy competing adhesion mechanisms to ensure successful colonization and dispersal in the host.

In summary, *S. aureus* cells use their unique adhesive mechanisms to colonize biotic surfaces in humans, rendering *S. aureus* a common opportunistic bacterial species in many contexts. Biofilm formation is closely associated with pathogenicity in *S. aureus*. For example, Parastan et al. reported a significant correlation between the adhesion-related genes and multidrug resistance patterns of *S. aureus* clinical isolates (Parastan et al., 2020). The MSCRAMMs and other less-understood adhesion mechanisms all help staphylococci invade the human body and evade the immune system. Therefore, a better understanding of the adhesion mechanism of *S. aureus* can facilitate the development of new drugs targeting the adhesins of *S. aureus* and inhibit its surface attachment during infection.

## Conclusion and Outlook

Much remains to be learned regarding how microbes develop their communal attachment to a wide range of foreign surfaces. Different species achieve biofilm adhesion through different mechanisms: In the examples reviewed here, *V. cholerae* possesses specific matrix proteins that, in conjunction with the exopolysaccharide, achieve adhesion of the entire biofilm. On the other hand, *P. aeruginosa* biofilms appear to use their major exopolysaccharide, jointly with pili, for surface adhesion. Finally, staphylococci exhibit multiple biofilm-forming strategies: Some strategies depend on exopolysaccharide and ECM-binding adhesins, while others depend on the formation of amyloid fibers. Indeed, it seems that the Aap/SasG- or Bap-dependent biofilm formation pathways are *orthogonal* to the exopolysaccharide-based strategy. This might not be unique to staphylococci; *V. cholerae* is also known to form VPS-independent biofilms (Kierek and Watnick, 2003; Müller et al., 2007). A central scheme that starts to emerge for biofilm adhesion is the dual function of the glue: The adhesion molecules need to attach to the biofilm-dwelling cells on one side, either through direct anchoring or indirectly through exopolysaccharide; on the other hand, the adhesin needs to attach to a foreign surface, be it a bare abiotic surface or a host surface decorated with specific molecules. It is unclear what the advantages and disadvantages of the different strategies are, partly due to our limited knowledge about the biochemical details of many of the adhesive molecules and their interactions with foreign surfaces.

Looking forward, we propose following research avenues to further enrich our understanding of biofilm adhesion:

(1) Structural biology: More crystal structures of the adhesins need to be solved to build our understanding of bacterial adhesion to specific biotic surfaces. The recent rapid progress in cryo-EM could be harnessed for structural solving (Cheng et al., 2015), and classical X-ray crystallography can provide high-resolution details of ligand binding motifs.
(2) Biophysical characterization: While AFM has become a powerful tool in the study of bacterial adhesion, significant technical expertise is required to perform and interpret AFM experiments. Closer collaboration between microbiologists and biophysicists will certainly lead to many new discoveries regarding bacterial adhesion.
(3) The connection between cell adhesion and biofilm architecture should be further explored in other species. So far, the single-cell biofilm imaging technique has mainly been applied to *V. cholerae* biofilms, revealing well-defined cell ordering that is highly dependent on cell-to-surface adhesion. It remains to be seen whether such cell ordering is a general phenomenon in all biofilm-forming species.
(4) Molecular scale information has yet to be connected with bulk adhesion measurements. Many techniques reviewed here reveal the adhesive energy between bulk biofilms (such as a colony biofilm) and a substrate; it is unclear how to compare AFM measurements at the cellular level to these bulk measurements.

We believe that with coordinated efforts from microbiologists, biophysicists, and engineers, the secret ingredients of biofilm adhesion will be uncovered in the near future. A comprehensive understanding of biofilm adhesion will pave the way for the discovery of new chemicals that specifically target biofilm-surface interactions and lead to more forward-looking, innovative functional biofilm materials that adhere to various surfaces on demand.

## Author Contributions

ZJ reviewed the literature and complied the review. TN, SM, RO, and JY revised the initial draft and reviewed the final draft. All authors contributed to the article and approved the submitted version.

### Conflict of Interest

The authors declare that the research was conducted in the absence of any commercial or financial relationships that could be construed as a potential conflict of interest.
